# Vaginal Perforations After Fecal Management System Use in Critically Ill Patients: A Case Series

**DOI:** 10.7759/cureus.102338

**Published:** 2026-01-26

**Authors:** Sarah V Sebastian, Nicole P Jenkins, Amber W Chan, Stephanie A Sansone, Scott Smilen

**Affiliations:** 1 Department of Obstetrics and Gynecology, Hackensack Meridian Jersey Shore University Medical Center, Neptune, USA; 2 Division of Urogynecology, Department of Obstetrics and Gynecology, Mount Auburn Hospital, Harvard Medical School, Boston, USA; 3 Division of Urogynecology, Department of Obstetrics and Gynecology, Hackensack Meridian Jersey Shore University Medical Center, Neptune, USA

**Keywords:** critically ill patients, fecal incontinence, fecal management system, vaginal perforation, vaginal trauma

## Abstract

Fecal incontinence is a relatively common condition in patients admitted to the intensive care unit (ICU). Fecal management systems (FMS) were developed to divert stool away from the skin. While they have benefits, rare but serious complications can occur. This case series discusses two cases of vaginal perforations following FMS use in critically ill patients. An 87-year-old female was admitted twice to the ICU for infective endocarditis and bacterial pneumonia. During her second ICU stay, an FMS device was in place for up to 19 days, after which a vaginal perforation with stool leakage from the vagina was noted. In the second case, a 34-year-old female was admitted following disseminated intravascular coagulation (DIC) from a likely amniotic fluid embolism. An FMS was in situ for four days before a vaginal perforation, with stool leakage from the vagina, occurred. In both cases, neither patient was stable for an exam under anesthesia with possible repair, and therefore conservative management was pursued. Vaginal perforations can occur with FMS, and healthcare workers should be aware of this possible complication. Critically ill patients are at greater risk due to comorbid conditions that may impair tissue integrity, lead to possible fistula formation, and impede successful repair. While currently not listed in manufacturer guidelines, coagulopathies, including DIC, may be considered a relative contraindication for FMS use. Like other medical devices, FMS should be monitored for correct indication and proper placement and be regularly reassessed.

## Introduction

Fecal incontinence (FI) affects approximately 10% of critically ill patients admitted to intensive care units (ICUs) [1]. ICU patients are at increased risk for large-volume liquid stools due to enteral feeds, hypoalbuminemia, changes in gut microflora, and medications such as antibiotics [2]. Sequelae of FI include incontinence-associated dermatitis, healthcare-associated pressure injuries (HAPI), fluid and electrolyte imbalances, infections, and emotional distress to patients and their families [3]. Traditionally, 20-30 French rectal tubes were inserted into the rectum to divert liquid stool, but require intermittent inflation and deflation of a high-pressure balloon to prevent rectal mucosal injury [3]. Fecal management systems (FMS), introduced in the early 2000s, divert stool from skin and medical equipment using a flexible catheter attached to a collection bag and held in place in the rectum with a low-pressure, saline-filled balloon [4]. These devices reduce the need for labor-intensive nursing care, decrease the risk of perianal skin breakdown, can be used to administer medications, and decrease transmission of fecal microbiota [3,4]. In contrast to a colostomy, which surgically diverts stool through an abdominal stoma [5], FMS maintain the native anorectal connection and provide a temporary, non-operative intraluminal method of stool diversion suitable for critically ill patients.

Known complications of FMS include rectal mucosa injury, gastrointestinal hemorrhage, and rectovaginal fistulae [6]. This case series discusses two cases of vaginal perforations that occurred in critically ill patients after use of an FMS during hospital admission. In both cases, surgical management was not initially pursued.

## Case presentation

Case 1

An 87-year-old female, para 1 (mode of delivery not documented), presented to the hospital after a two-day course of generalized weakness and confusion. Past medical history included vaginal intraepithelial neoplasia (VAIN) 3, atrial fibrillation on apixaban 5 mg twice daily, heart failure with reduced ejection fraction (HFrEF), chronic obstructive pulmonary disease (COPD) on home oxygen of 4 L/minute nasal cannula, type 2 diabetes mellitus, and hypertension. Past surgical history included supracervical hysterectomy with bilateral salpingo-oophorectomy (>45 years prior) and CO_2_ laser ablation of the upper vagina (six years prior). The patient was followed by gynecology oncology for VAIN 3 and known to be in remission without residual disease burden.

By exam, labs, and imaging, she met the criteria for sepsis. She was admitted, subsequently diagnosed with infective endocarditis, and received prolonged courses of intravenous antibiotics. On hospital day nine, the patient was transferred to the medical intensive care unit (MICU) for hypoxic hypercapnic respiratory failure with intubation and hypotension requiring pressors.

On hospital day 15, the patient developed hematochezia with anemia requiring transfusion with two units of packed red blood cells (pRBCs). Computed tomography (CT) angiogram of the abdomen/pelvis showed an active gastrointestinal bleed of the posterior rectum with a large stool burden and mild diffuse rectal wall thickening, suggesting stercoral proctitis. Colonoscopy was deferred as hemoglobin rose appropriately without further intervention. She was then started on heparin for anticoagulation. On hospital day 45, the patient was re-admitted to the MICU for respiratory failure from superimposed bacterial pneumonia.

During her second ICU admission, she developed fecal incontinence and multiple HAPI. An FMS was placed and apparently in situ for up to 19 days after the second ICU re-admission date, although documentation of placement was not clear.

**Figure 1:**
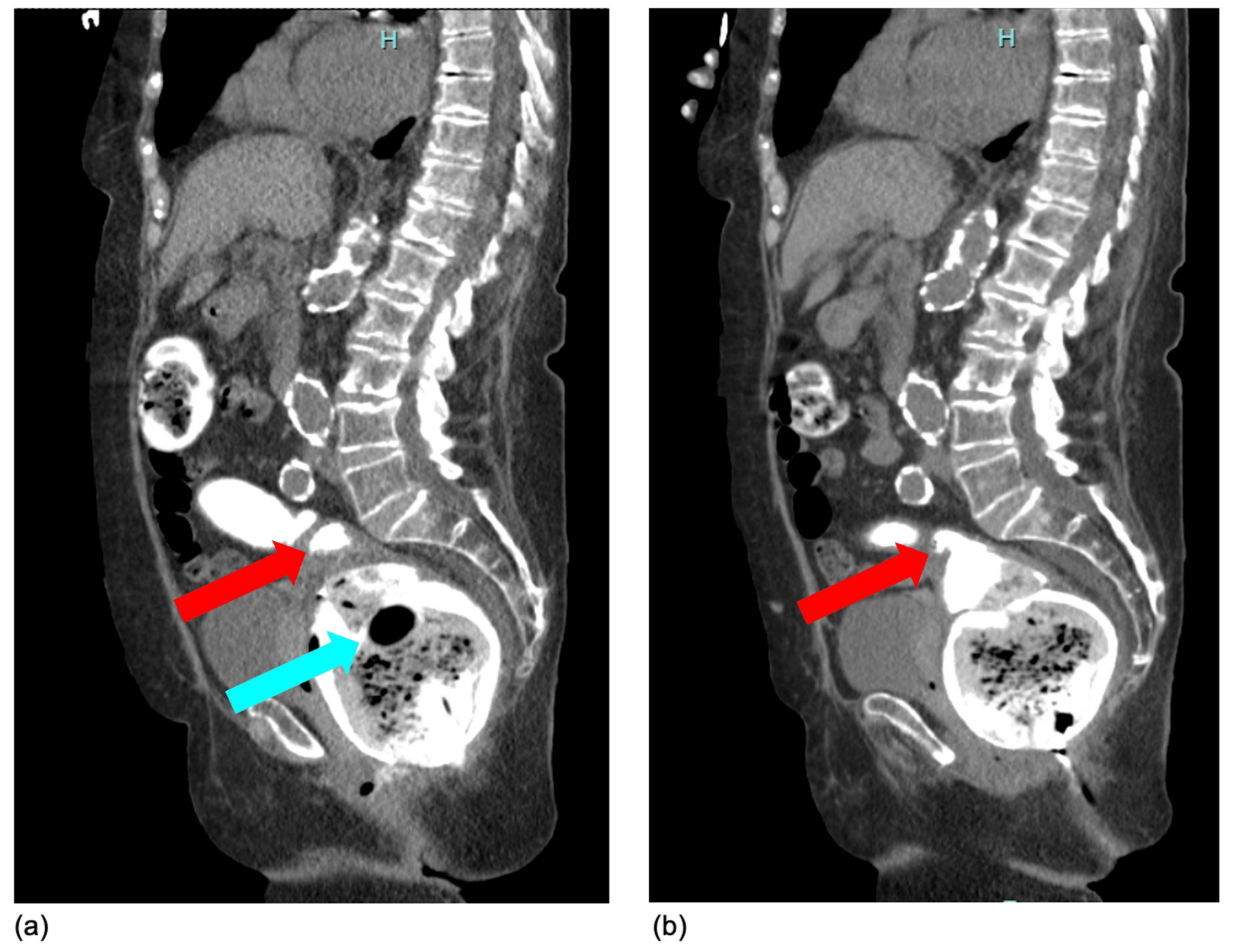
Sagittal views of computed tomography of the abdomen/pelvis for Case 1 Sagittal views of computed tomography (CT) of the abdomen/pelvis with rectal contrast on hospital day 65, after up to an estimated 19 days of FMS use, before (a) and after (b) evacuation of the rectum, showing focal anterior extension of enteric contrast at the level of the rectum towards the vagina. Red arrow = area of focal contrast; blue arrow = rectal tube

On hospital day 63, gynecology and colorectal surgery were consulted for stool passage from the vagina. On physical exam, light brown fluid was seen coming from between the labia, but the patient could not tolerate a full exam in her ICU bed. CT abdomen/pelvis with rectal contrast on hospital day 65 revealed a focal anterior extension of enteric contrast at the level of the rectum extending towards the vagina with persistence after evacuation (see Figure 1). She was noted to have a severely distended rectum with a rectal tube in situ.

Following identification, the rectal tube was promptly removed, and the vaginal perforation site was identified within the proximal rectum, thus yielding a recommendation for a diverting colostomy rather than primary vaginal repair. Given the patient's multiple comorbidities, poor prognosis, and inability to follow commands, a goals-of-care discussion was held, and the family decided to pursue comfort care only. The patient was discharged home on hospital day 74.

Case 2

A 34-year-old female, para 8 (uncomplicated vaginal deliveries), presented for dilation and evacuation of an intrauterine fetal demise measuring 16.3 weeks on ultrasound and 22.6 weeks by last menstrual period. Her past medical history was notable for a BMI of 30 and a past surgical history of an appendectomy.

Dilation and evacuation of products of conception was performed in the usual manner under ultrasound guidance with complete removal and no intra-operative complications. Immediately post-procedure, she had a spontaneous onset of cardiovascular collapse from a suspected amniotic fluid embolism, which required cardiopulmonary resuscitation and admission to the MICU.

While in the MICU, she progressed into disseminated intravascular coagulation (DIC) requiring 32 units of pRBCs, 14 units of fresh frozen plasma, seven units of platelets, and two units of cryoprecipitate. Vaginal bleeding was controlled with an intrauterine tamponade balloon, misoprostol of 1,000 mcg per rectum, methylergonovine of 0.2 mg intramuscular, and tranexamic acid of 1 g intravenously. During this pelvic exam, no uterine perforation or vaginal trauma/perforation was identified. Due to ongoing resuscitation with massive transfusion protocol, a CT scan was performed, which demonstrated a large subcapsular liver hematoma with active extravasation consistent with a liver laceration. She underwent an emergent exploratory laparotomy and repair of the liver laceration.

**Figure 2:**
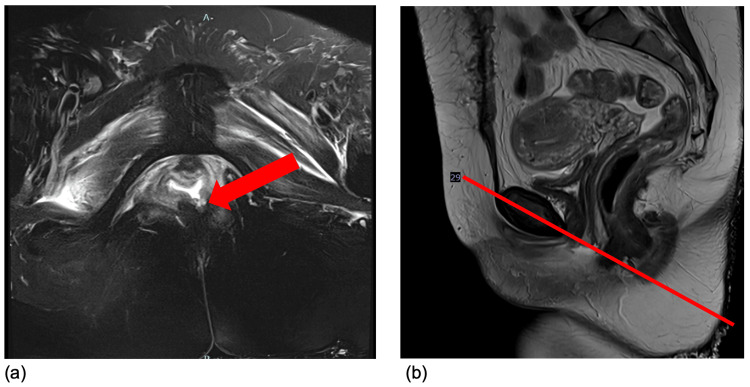
Magnetic resonance imaging (MRI) of the rectum for Case 2 Oblique (a) and sagittal (b) views of magnetic resonance imaging (MRI) of the rectum with and without contrast on hospital day 19, after four days of FMS use, showing fluid in the inferior vagina leaking into the rectum through the posterior wall towards the anal sphincter. Red arrow = area of defect. Red line = corresponding anatomical location of the fistula on sagittal imaging

On hospital day six, a code stroke was called with magnetic resonance imaging (MRI) of the head, revealing multiple acute infarcts. Due to the patient’s acute neurologic change, intubation status, and large-volume diarrhea, an FMS was placed. On hospital day 13, the same day she was extubated and weaned off sedation, stool was noted to be leaking from the vagina. The rectal tube, in situ for a total of four days, was removed at this time. Thin, loose stool was seen along the entire aspect of the vulva, but no defects were appreciated on a limited digital rectal and vaginal exam in her ICU bed. Barium enema showed visualization of contrast within the vagina during fluoroscopy. On MRI rectum with and without contrast, a rectal defect was illustrated in the left posterior vagina wall, appearing to connect with the anal canal (see Figure 2).

An exam under anesthesia and primary vaginal repair was recommended. However, due to concern for poor surgical outcome given deconditioning, malnutrition, and overall stress response from major abdominal surgery, the recommendation was an interval exam under anesthesia. At a gynecology follow-up visit nine months after her first hospitalization, delayed due to three interim re-admissions, there was no further leakage of stool nor a fistulous tract identified on exam. Since initial admission, the patient developed end-stage renal disease on hemodialysis and demonstrated residual left-sided weakness from her cerebrovascular accident during hospitalization.

The timeline of complications from FMS in both patients is presented in Table 1.

**Table 1:** Timeline of fecal management system complications in this case series For all columns, the number of days is counted from day one of hospitalization.

Case No.	Fecal Management System Insertion	Appearance of Symptoms	Confirmation of Vaginal Perforation on Imaging
Case 1	Day 44-Day 62	Day 63	Day 65
Case 2	Day 9	Day 13	Day 19

## Discussion

These cases demonstrate two instances of vaginal perforations or trauma due to the utilization of FMS.

Comorbidities contributing to delayed wound healing in critically ill patients

Patients in the ICU often have co-morbidities, which, when combined with the pressure exerted on the rectal mucosa and rectovaginal septum by the balloon of an FMS, could increase risk for local tissue damage. Wound healing relies on hemostasis, inflammation, proliferation, and scar maturation, all of which systemic illness may disrupt [7]. Coagulopathies, such as DIC, can significantly impair wound healing by depleting vital clotting factors and platelets, resulting in an increased risk of bleeding and failure to form stable clots at wound sites [8]. Compared to trauma-induced coagulopathies, which often subside as the acute injury resolves with supportive care, DIC associated with critical illness or infection creates a sustained imbalance in coagulation and fibrinolysis [9]. Prolonged impairment of the coagulation/fibrinolytic balance in DIC increases the risk of both persistent bleeding and poor tissue healing, posing a particular challenge when a foreign device is in situ.

Further compounding this risk, comorbid cardiovascular disease and chronic hypoxia impair tissue perfusion and oxygenation. Surgical wounds or device-related mucosal disruption are more likely to progress to chronic, non-healing wounds in the context of impaired immunity, ongoing microvascular coagulation, and poor tissue oxygenation [7]. Hypoperfusion and hypoxia associated with shock or cardiovascular disease, directly and via reduced angiogenesis, further delay wound healing by interfering with granulation and re-epithelialization [10]. Thus, such patients are uniquely susceptible to pressure-induced injuries, especially in the setting of prolonged exposure to a retained device such as FMS.

DigniShield^TM^ stool management system

The DigniShield^TM^ Stool Management System (SMS) (C.R. Bard, Inc., Covington, GA), the device used in both cases, is designed for fecal management in immobilized or critically ill patients to minimize skin exposure to stool and reduce the risk of skin breakdown [11]. This device is placed via the rectum and utilizes an internal balloon for retention, along with interconnected tubing systems for sampling, flushing, and closed drainage. The manufacturer’s instructions highlight patient selection, proper patient positioning with tube placement, routine irrigation, and frequent assessments to confirm the device remains in the appropriate position and functions. The maximum time the DigniShield^TM^ SMS may remain in place is 29 consecutive days [11]. In both cases, documentation confirming patient selection, placement technique, balloon volume, and re-assessments is unavailable. This underscores the importance of these steps in preventing iatrogenic harm that can develop from using FMS.

Contraindications include patients with fecal impaction, lower large bowel or rectal surgery within the last year, rectal or anal injury, severe rectal or anal stricture or stenosis, rectal or anal tumors, severe hemorrhoids, or significant impairment of rectal mucosa (i.e., severe or ischemic proctitis, mucosal ulcerations) [11]. Notably, current manufacturer guidelines do not list coagulopathy as a relative contraindication. This omission highlights the potential risk of using the DigniShield^TM^ system in patients with systemic coagulopathy when no clear guidance or warning is provided. DigniShield^TM^ is designed similarly to other FMS devices on the market [4], and complications have occurred with multiple types of FMS [6,12-18]. The risk of rectovaginal injury likely reflects a class effect related to device design, dwell time, and patient factors rather than a single product; therefore, providers should avoid using any FMS device in patients with systemic coagulopathies.

**Figure 3:**
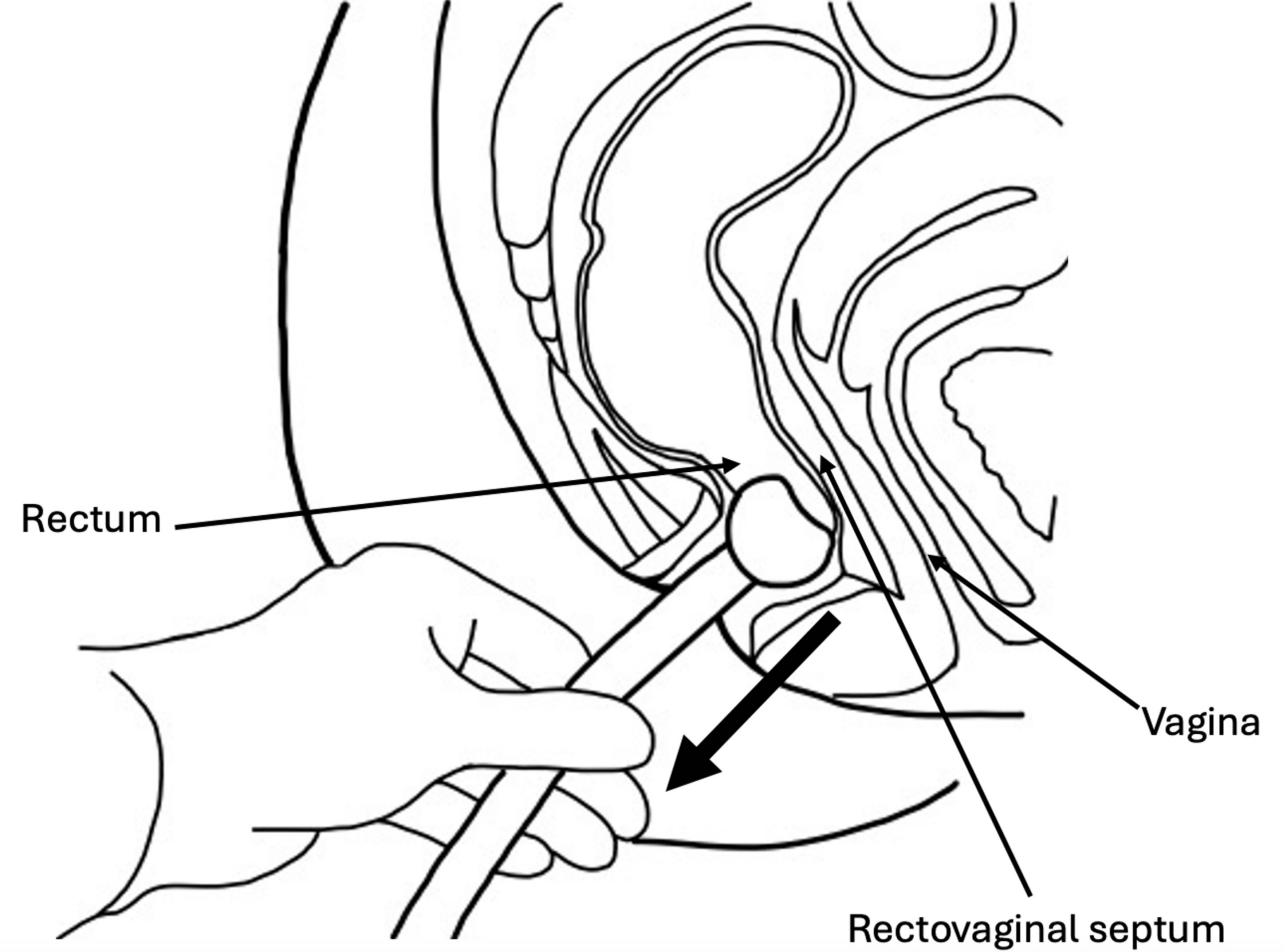
Placement of a fecal management system in relation to the rectum and vagina Sagittal view illustrating placement of a fecal management system (FMS), followed by gentle traction on the drainage tube (large downward arrow) to confirm correct placement of the FMS balloon against the rectal floor. Note the placement of the FMS balloon within the rectum and its proximity to the rectovaginal septum and the vagina.

When properly placed, the rectal balloon of FMS sits against the floor of the rectum. However, due to the proximity of the balloon to the rectovaginal septum and vagina, as illustrated in Figure 3, in a critically ill patient, the pressure exerted by the balloon could contribute to vaginal perforations, as seen in this case series.

Adverse effects of fecal management systems

Like any medical device, FMS has associated risks. Although the incidence of rectal complications from FMS was 1.3% in a single-institution, retrospective cohort study, most cases required surgical/endoscopic intervention with high morbidity and mortality [6]. Similarly, low rates of hematochezia (1.5%) were reported in a study of ICU patients; one patient was managed conservatively, but the other required ligation of rectal mucosal bleeding [12]. There are also case reports of massive gastrointestinal hemorrhage associated with FMS [13,14]. A small randomized controlled trial (n=79) observed anal erosions in about 12% of patients [15]. However, another small prospective cohort study of patients with FMS devices (n=20) found none with evidence of rectal injury on proctoscopy [16]. In another retrospective study (n=50), 26% of patients experienced complications from FMS, including over-inflation of the balloon without rectal mucosal injury (14%), temporary anal atony (8%), and stool leakage around the catheter (4%), but no rectal mucosal injuries or fistulae [17]. In the Kane et al. study, complications occurred after a median of seven days (range: 1-22 days), with half of them occurring within three days following placement [6]. Therefore, healthcare workers should be aware that adverse effects can occur quickly after placement. Institutional protocols for FMS use exist, such as the one developed by Kane et al. [6].

There are three reported cases of rectovaginal fistulas following FMS use. In the first case, a 66-year-old female patient who underwent FMS placement during a prior admission for diabetic ketoacidosis presented with three months of fecal incontinence. A 2.5 cm ano-vaginal fistula was identified, and a diverting sigmoid colostomy was created [18]. In the second case, an 18-year-old female patient with T-cell acute lymphoblastic leukemia status post chemoradiotherapy and hematopoietic stem cell transplant was admitted for hepatic encephalopathy. FMS was placed due to diarrhea from rifaximin and lactulose, and a rectovaginal fistula developed one month later. Unfortunately, this patient was not medically stable enough to undergo surgical repair and died before corrective measures could be taken [19]. The third case occurred in an elderly female patient who suffered multi-system trauma and had pressure necrosis from long-term FMS use, but no further details are available [20]. These cases are summarized in Table 2.

**Table 2:** Reported cases of rectovaginal fistula following fecal management system use Case details and outcomes of three reported cases of rectovaginal fistula associated with fecal management system (FMS) use

Case	Patient Details	Indication for Fecal Management System (FMS)	Duration of FMS Use	Fistula Characteristics	Management & Outcome
Massey et al., 2010 [18]	66-year-old female with recent intensive care unit (ICU) admission for diabetic ketoacidosis (DKA), presenting with three months of fecal incontinence	Management of diarrhea during DKA hospitalization	Exact duration not reported; fecal incontinence occurred after prior hospitalization with symptoms noted months later	2.5 cm ano-vaginal fistula with complete anal sphincter disruption; fistula biopsy consistent with ischemic trauma	Underwent diverting sigmoid colostomy for symptomatic control; uneventful postoperative recovery and hospital discharge
Butts et al., 2019 [19]	18-year-old female with T-cell acute lymphoblastic leukemia, post-allogenic stem cell transplant, hepatic encephalopathy with multi-organ failure	Management of diarrhea from rifaximin and lactulose	31 days	~2 cm rectovaginal fistula, rectal tube opening palpable in vagina	Surgical repair planned but not performed due to instability; patient died two days later from cardiac arrest, presumed from septic shock
Bauer et al., 2024 [20]	Female in her 60s with multisystem trauma, prolonged ICU stay	Long-term stool containment during protracted critical illness	Prolonged use; exact duration not reported	Rectovaginal fistula due to pressure necrosis from extended FMS use	Underwent loop sigmoid colostomy; discharged to extended care facility

In both patients in our series, vaginal perforations occurred shortly following FMS placement, between four and nineteen days. This is consistent with the results observed by Kane et al., as well as other case reports and small studies [6,12-14]. Development of vaginal perforations is concerning, as vaginal trauma is a risk factor for rectovaginal fistula [5], which has occurred after FMS use. Furthermore, ICU patients who develop a perforation may be too unstable for prompt recognition and repair of injury.

These findings highlight an important distinction between acute vaginal perforation and established rectovaginal fistula. In our two patients, their imaging and clinical course were most consistent with an acute or subacute defect in the rectovaginal septum that was identified shortly after the onset of symptoms, whereas previously reported cases describe tracts presenting later and often required diversion or complex repair [17-19]. Thus, early recognition and removal of the FMS in the setting of a new perforation may prevent progression to a mature rectovaginal fistula in surviving patients, as appears to have occurred in our second patient.

## Conclusions

FMS are generally safe and provide benefits for critically ill patients. However, they are not risk-free. These patients developed vaginal perforations after FMS use, which should be included among known complications from FMS devices. Their underlying comorbidities may have been contributing factors. While not currently listed as a contraindication, coagulopathies should be considered a relative contraindication for FMS use. Like any other medical device, the indication for use and reassessment for continued use should be clearly documented in the medical record. Protocols for FMS use should be considered to increase patient safety.
